# Isoleojaponin, a New Halimane Diterpene Isolated from *Leonurus japonicus*

**DOI:** 10.3390/molecules20010839

**Published:** 2015-01-07

**Authors:** Hankui Wu, Sensheng Wang, Zhiyong Xu, Shanshan Sun, Haijuan Liu, Jinjin Wang, Yan E, Yanyan Lv, Xuelin Dong, Gang Li, Liping Zhang, Yunfeng Shi

**Affiliations:** College of Chemistry and Chemical Engineering, Anyang Normal University, Anyang 455000, Henan, China; E-Mails: wssn2006@163.com (S.W.); xzytyyl1314@hotmail.com (Z.X.); sssaynu@163.com (S.S.); liuaynu@163.com (H.L.); wangaynu@163.com (J.W.); yanaynu@163.com (Y.E.); lvaynu@163.com (Y.L.); dongaynu@163.com (X.D.); lpzhang70@163.com (L.Z.)

**Keywords:** *Leonurus japonicus*, labdane diterpenoid, isoleojaponin, halimane

## Abstract

Leojaponin (**2**), a labdane diterpene, was isolated from the EtOH extract of the herb of *Leonurus japonicus* together with a new halimane diterpene named isoleojaponin (**1**). Isoleojaponin has a new diterpene skeleton with a unique cross-conjugated α,β-unsaturated ketone system, Their structures were elucidated by physical and spectroscopic analysis, and the relative configuration of the chiral C-9 carbon was determined by a computational method, and analysis of its possible biogenesis pathways.

## 1. Introduction

**Figure 1:**
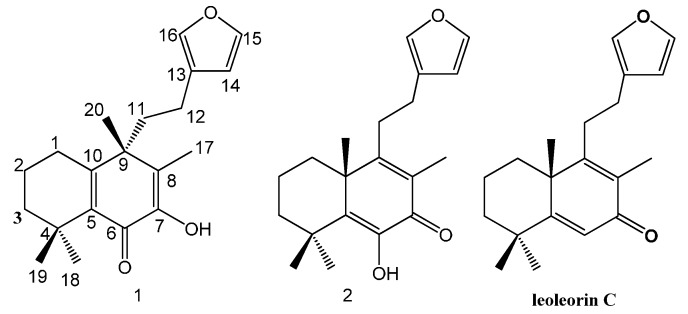
Structures of compounds **1**–**2** and leoleorin C.

*Leonurus japonics* Houtt. (Lamiaceae) is an annual or biennial herbaceous plant widely distributed and cultivated in China. The dried herb is used in Traditional Chinese Medicine for the treatment of various diseases, especially menstrual disturbances, dysmenorrhea, and amenorrhea [1]. Recently, phytochemical studies on this plant have been reported [2,3,4,5,6,7,8,9,10]. Our previous investigation on two plants of the family of *Lamiaceae* resulted in the isolation of a number of new labdane diterpenes [11,12]. In our research on psychoactive natural products from *L. japonicus*, two diterpenoids were isolated and identified. One of them was identified as leojaponin by comparison of the NMR data with those reported [2]. The other one was elucidated as its isomer and named isoleojaponin (**1**), it has transformed structure skeleton compared with a labdane-type compound such as leojaponin, the methyl group (Me-20) connected to C-10 has shifted to link with C-9, and the double bonds of ring-B were rearranged correspondingly. This paper describes the isolation and structure elucidation of these isolates (Figure 1).

## 2. Results and Discussion

**Figure 2:**
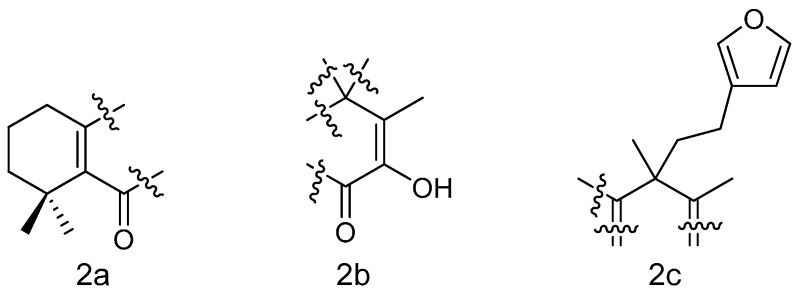
Partial structure of compound **1**.

Compound **1** was obtained as a light yellow oil. The molecular formula was C_20_H_26_O_3_ which was confirmed by analysis of NMR spectra (Table 1) and the HRESIMS [M+H]^+^ 315.1974 (calcd. 315.1960). Twenty carbon signals were observed including five methylenes, and four tertiary methyl groups as indicated by ^1^H-NMR and DEPT spectra (δ_H_ 1.25, 1.33, 1.35, 1.94, all *s*; δ_C_ 25.6, 28.3, 27.9, 11.2). There were nine *sp*^2^ carbons corresponding to two tetrasubstituted olefins and a monosubstituted furan ring, which was revealed by typical proton signals at δ_H_ 6.18 (s), 7.14 (s), 7.33 (d, *J* = 1.6 Hz) and the carbon signals at δ_C_ 110.7, 138.5, and 142.8. One of the tetrasubstituted alkenyl portions appeared to be part of an α, β-unsaturated ketone as revealed by the low field signals at δ_C_ 161.9, 180.1. In the HMBC spectrum, correlations of H-1/C-2, C-3, C-5, C-9, and C-10; H-3/C-2, C-4, C-5, C-18, C-19; H_3_-19 and H_3_-20/C-3, C-4, C-5, in combination with the obvious proton NMR pattern (δ_H_ 2.40 m, 2.25 m), ^1^H-^1^H COSY spectrum and carbon resonances, partial structure could be deduced as shown (Figure 2a).

**Table 1:** ^1^H-NMR and ^13^C-NMR data of **1** and **2** (400 and 100 MHz; CDCl_3_, δ ppm, *J* in Hz).

**Position**	**1**		**2**	
	δ_C_	δ_H_	δ_C_	δ_H_
1	27.8	2.24 m; 2.40 m m	29.4	1.55 m; 2.06 m
2	19.0	1.67 m	17.2	1.70 m; 1.88 m
3	41.3	1.55 m	37.2	1.42 m; 1.87 m
4	33.8		35.6	
5	138.5		138.6	
6	180.1		143.1	
7	145.0		181.6	
8	128.7		127.3	
9	45.7		165.8	
10	161.9		43.8	
11	37.7	1.92 m	31.5	2.57 m; 2.66 m
12	19.8	1.94 m	23.7	2.57 m
13	124.3		124.3	
14	110.7	6.18, s	110.5	6.34 s
15	142.8	7.33 d (1.6)	143.1	7.40 d (1.6)
16	138.6	7.14 s	138.6	7.30 s
17	11.2	1.94 s	11.5	1.94 s
18	28.3	1.33 s	28.0	1.35 s
19	27.9	1.35 s	27.6	1.36 s
20	25.6	1.25 s	27.9	1.32 s
OH		6.86 s		7.01 s

The signal of a hydroxyl group was observed, as the HMQC spectrum showed no correlation between the signal of hydroxyl proton (δ_H_ 6.86 *s*) and any other carbon, and the HMBC spectrum showed the correlations of the hydroxyl proton with three *sp*^2^ carbons (δ_C_, 145.0, 128.7, and 180.1), and also the HMBC correlations of H_3_-17 with C-7, C-8, and C-9 allowed to deduce another moiety as shown (Figure 2b). Correlations of H_3_-20/C-8, C-9, C-10, and C-11; H-11/C-8, C-9, C-10, C-12, C-20; H_2_-12/C-9, C-11, C-13, C-14 and C-16 suggested Me-20 was connected to a quaternary carbon which linked to two β-olefinic carbons of the cross conjugated α, â-unsaturated ketone and also linked to an ethyl group bearing a monosubstituted furanyl ring at its end (Figure 2c). To assemble the above deduced moieties, the planar structure of **1** was determined as 4-(2-(furan-3-yl)ethyl)-2-hydroxy-3,4,8,8-tetramethyl-5,6,7,8-tetrahydronaphthalen-1(4*H*)-one, which was named isoleojaponin.

**Figure 3:**
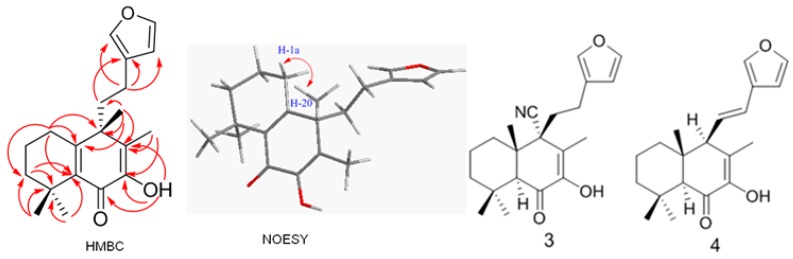
Selected HMBC and NOESY correlations of **1**, and structures of **3** and **4**.

**Figure 4:**
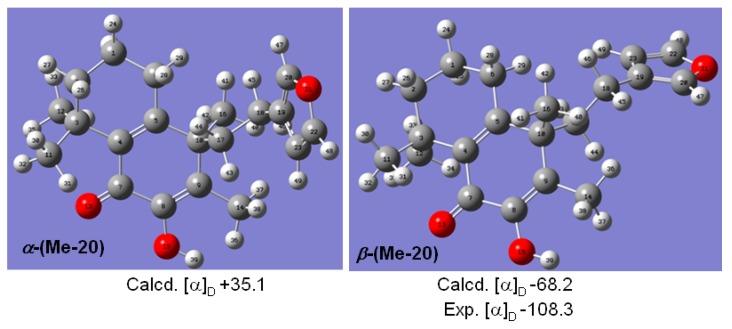
The computational and experimental OR values for α and β-(Me-20) epimers.

Considering the stereochemistry of **1**, several similar structures (Figure 3) have been reported [13]: 9α-cyano-15,16-epoxy-7-hydroxylabda-7,13(16),14-trien-6-one (**3**) and 7-hydroxyhedychenone (**4**) have the same configuration at C-9, and an X-ray structural analysis of **3** has confirmed the C-9α position of the nitrile group. However, the labdane-type diterpeniods **3** and **4** have no similar cross-conjugated systems as **1**, so the stereochemistry of **1** could not be decided by simple comparison with the reference pattern. In order to investigate the relative configuration of C-9, computation of specific optical rotation was used in this study, the model of both α and β Me-20 epimers were built, and the optical rotation values were obtained at the B3LYP/6-31+g (d) level in methanol via PCM model with Gaussian 09. The results were shown (Figure 4). We found that the computational specific optical rotation value for α-(Me-20) epimer was +35.11, and for the β-(Me-20) was −68.23. Comparison with experimental result ${\left[\alpha \right]}_{D}^{20}$ = −108.3 (in MeOH), compound **1** should have an β-orientation for Me-20 as indicated by the negative values of both experimental and computational optical rotation.

**Figure 5:**
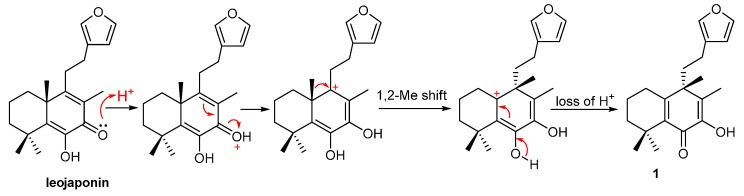
Proposed formation of **1** from leojaponin via 1,2-Me shift.

The relative stereochemistry of compound 1 could not be completely established by application of NOE experiments, because the NOESY correlations between H_2_-1 and H_3_-20 could not determine α or β-orientation for Me-20, although the cross peak was observed (Figure 3). However, it would be reasonable to deduce from the biogenesis pathways of the co-isolation of compound **1** and **2** (with definitely β-orientation for Me-20), a stereospecific 1,2-Me migration might result in the formation of **1** as shown (Figure 5), and this type of reaction was observed by biomimetic rearrangements of simplified labdane diterpenoids [14]. Therefore, compound **1** was identified as (*S*)-4-(2-(furan-3-yl)ethyl)-2-hydroxy-3,4,8,8-tetramethyl-5,6,7,8-tetrahydro-naphthalen-1(4*H*)-one, and named isoleojaponin.

Compound **2** was isolated as a yellowish oil. The ^1^H-NMR and ^13^C-NMR spectra of **2** were different from those of **1** (Table 1) and leoleorin C [11], but almost identical to those of leojaponin [2], so **2** was identified as leojaponin.

## 3. Experimental Section

### 3.1. General

NMR spectra were recorded on a Bruker Avance III spectrometer operating at 400 MHz for ^1^H- and 100 MHz for ^13^C-HRESIMS were measured with Waters UPLC-LCT Premier XE and was controlled by MassLynx 4.1 software. Optical rotations were acquired with a WZZ-2ss automatic polarimeter (Shanghai Shenguang High Strength Bolts Co., Ltd, Shanghai, China). UV spectra were recorded on a Quawell Q5000 UV/Vis spectrophotometer. IR spectra were recorded on a Varian 800 FT-IR spectrophotometer. Column chromatography was performed with silica gel (200–300 mesh, Yantai Institute of Chemical Technology, Yantai, China), Sephadex LH-20 (GE Healthcare Bio-Sciences AB, Uppsala, Sweden). HPLC separation was performed on an instrument (LC-3000, Beijing Chuangxintongheng Science & Technology Co., Ltd, Beijing, China) consisting of two pumps and a UV/Vis detector with an YMC-ODS-A (250 × 10 mm) semi-preparative column packed with C_18_ (5 μm).

### 3.2. Plant Material

The herb of *Leonurus japonicus* was purchased from Zhangye, Gansu Province of China in March, 2014. It was identified by one of the authors (Dr. H. Wu). A voucher specimen (Code: hkwu-aynu-20140301) was deposited at the Lab of Pharmaceutical Research, Anyang Normal University.

### 3.3. Extraction and Isolation

The dried and powdered herb of *L. leonurus* (85 kg) was extracted with 95% EtOH (3 × 400 L) by percolation at room temperature. The solvent was evaporated under reduced pressure at 45 °C to yield 7.2 kg viscous syrup, which was dissolved in water and extracted with EtOAc to get 3 kg extract. The EtOAc fraction was suspended in 30% EtOH and allowed to pass through a column (30 cm × 100 cm) packed with AB-8 macroporous resin (10 kg), then eluted with 50%, 70%, 90%, and 95% EtOH to obtain fractions A–D. Fraction C (693 g) was separated by silica gel CC over petroleum ether–EtOAc (10:1, 4:1, 2:1, 1:1) to yield subfractions (C1–C4). The further separation of fraction C1 with Sephadex LH-20 (MeOH), silica gel CC petroleum ether–EtOAc (80:1) to yield a mixture of **1** and **2** (11 g). 100 mg of the mixture was separated by reversed-phase semipreparative HPLC (95% MeOH in water) to yield pure **1** (32 mg) and **2** (50 mg).

*Isoleojaponin* (**1**): Light yellow oil, ${\left[\alpha \right]}_{D}^{20}$ = −108.3 (*c* = 0.1, MeOH); ^1^H- and ^13^C-NMR, see Table 1, HR-ESIMS *m/z* 315.1974 [M+H]^+^ (calcd for C_20_H_26_O_3_, 315.1960). IR (KBr) ν_max_: 1716, 1692, 1598, 1451, 1062, 1029 cm^−1^. UV (MeOH) λ_max_ nm (log ε): 260 (3.5), 307 (3.2).

## 4. Conclusions

The EtOH extract of the herb of *L. leonurus* was purified by multiple chromatographic methods. From the oily mass of one fraction two diterpeniods were isolated and identified. One was leojaponin, which can protect primary cultured rat cortical cells from glutamate-induced toxicity [6]. The new compound is isoleojaponin, bearing a furanyl group and a unique cross-conjugated á,â-unsaturated ketone system with -OH near the carbonyl group. To the best of our knowledge, diterpenes with these constructs are unprecedented and the formation of isoleojaponin from leojaponin might be caused by a cascade carbocation rearrangement whereby leojaponin underwent a 1,2-Me shift of Me-20, and two double bonds migrated and the positions of the carbonyl and hydroxyl groups exchanged correspondingly (Figure 5).

## Supplementary Materials

Supplementary materials can be accessed at: http://www.mdpi.com/1420-3049/20/01/0839/s1.

## References

[1] Liu Z.K., Wu D.R., Shi Y.M., Zeng T., Liu S.H., Du X., Dang Y.J., Xiao W.L., Sun H.D. (2014). Three new diterpenoids from *Leonurus japonicus*. Chin. Chem. Lett..

[2] Romero-González R.R., Avila-Núñez J.L., Aubert L., Alonso-Amelot M.E. (2006). Labdane diterpenes from* Leonurus japonicus* leaves. Phytochemistry.

[3] Ye M., Xiong J., Zhu J.J., Hong J.L., Zhao Y., Fan H., Yang G.X., Xia G., Hu J.F. (2014). Leonurusoleanolides E-J, minor spirocyclic triterpenoids from *Leonurus japonicus* fruits. J. Nat. Prod..

[4] Fuchino H., Daikonya A., Kumagai T., Goda Y., Takahashi Y., Kawahara N. (2013). Two new labdane diterpenes from fresh leaves of* Leonurus japonicus* and their degradation during drying. Chem. Pharm. Bull..

[5] Chang J.M., Shen C.C., Huang Y.L., Shieh B.J., Chen C.C. (2010). Two new glycosides from *Leonurus japonicus*. J. Asian Nat. Prod. Res..

[6] Moon H.I. (2010). Three diterpenes from *Leonurus japonicus* Houtt protect primary cultured rat cortical cells from glutamate-induced toxicity. Phytother. Res..

[7] Xiong L., Peng C., Zhou Q.-M., Wan F., Xie X.-F., Guo L., Li X.-H., He C.-J., Dai O. (2013). Chemical Composition and antibacterial activity of essential oils from different parts of *Leonurus japonicas* Houtt. Molecules.

[8] Zhang Y., Deng S., Qu L., An Y.-T., Wu C.-H., Han L.-F., Gao X.-M., Wang T. (2013). Rare syringyl acylated flavonol glycosides from the aerial parts of *Leonurus japonicas* Houtt. Molecules.

[9] Xiong L., Zhou Q.-M., Peng C., Xie X.-F., Guo L., Li X.-H., Liu J., Liu Z.-H., Dai O. (2013). Sesquiterpenoids from the herb of *Leonurus japonicus*. Molecules.

[10] Peng F., Xiong L., Zhao X.-M. (2013). A bicyclic diterpenoid with a new 15,16-dinorlabdane carbon skeleton from *Leonurus japonicas* and its coagulant bioactivity. Molecules.

[11] Wu H., Li J., Fronczek F.R., Ferreira D., Burandt C.L., Setola V., Roth B.L., Zjawiony J.K. (2013). Labdane diterpenoids from *Leonotis leonurus*. Phytochemistry.

[12] Wu H., Fronczek F.R., Ferreira D., Burandt C.L., Zjawiony J.K. (2011). Labdane diterpenoids from *Leonurus sibiricus*. J. Nat. Prod..

[13] Van Wyk W.W., Gray C.A., Keyzers R.A., Rivett D.E.A., Caira M.R., Nader B.S., Davis G.E., Werk T.L., Coleman M.T.D. (2005). Transformations of hispanolone. Novel Michael adducts with in planta activity against rice blast. Tetrahedron.

[14] George J.H., McArdle M., Baldwin J.E., Adlington R.M. (2010). Biomimetic rearrangements of simplified labdane diterpenoids. Tetrahedron.

