# Supplementary material for: Isoleojaponin, a New Halimane Diterpene Isolated from Leonurus japonicus
Source: Molecules. 2015 Jan 7;20(1):839–45. doi: 10.3390/molecules20010839 (PMC6272656; doi:10.3390/molecules20010839)
Supplement: Supplementary file 1 [file molecules-20-00839-s001.pdf]

Supplementary Materials

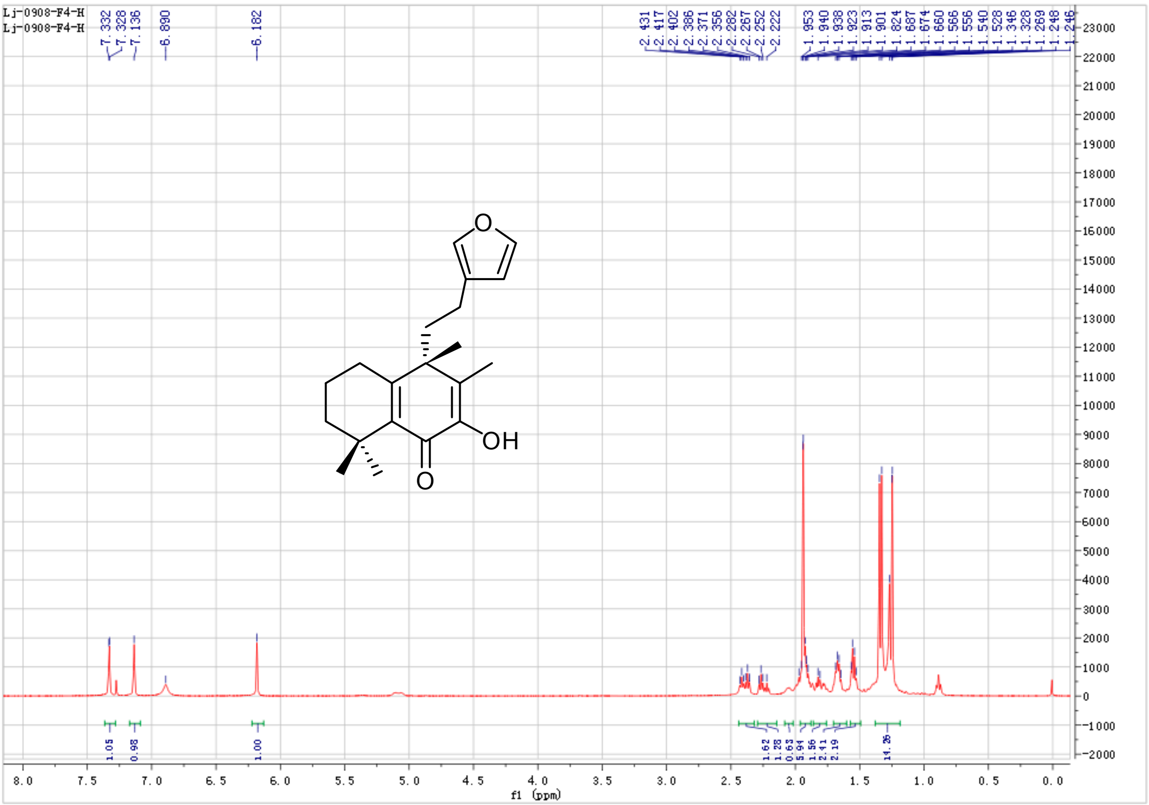

Figure S1. ^{1}H-NMR spectrum for isoleojaponin (**1**) in $CDCl_3$.

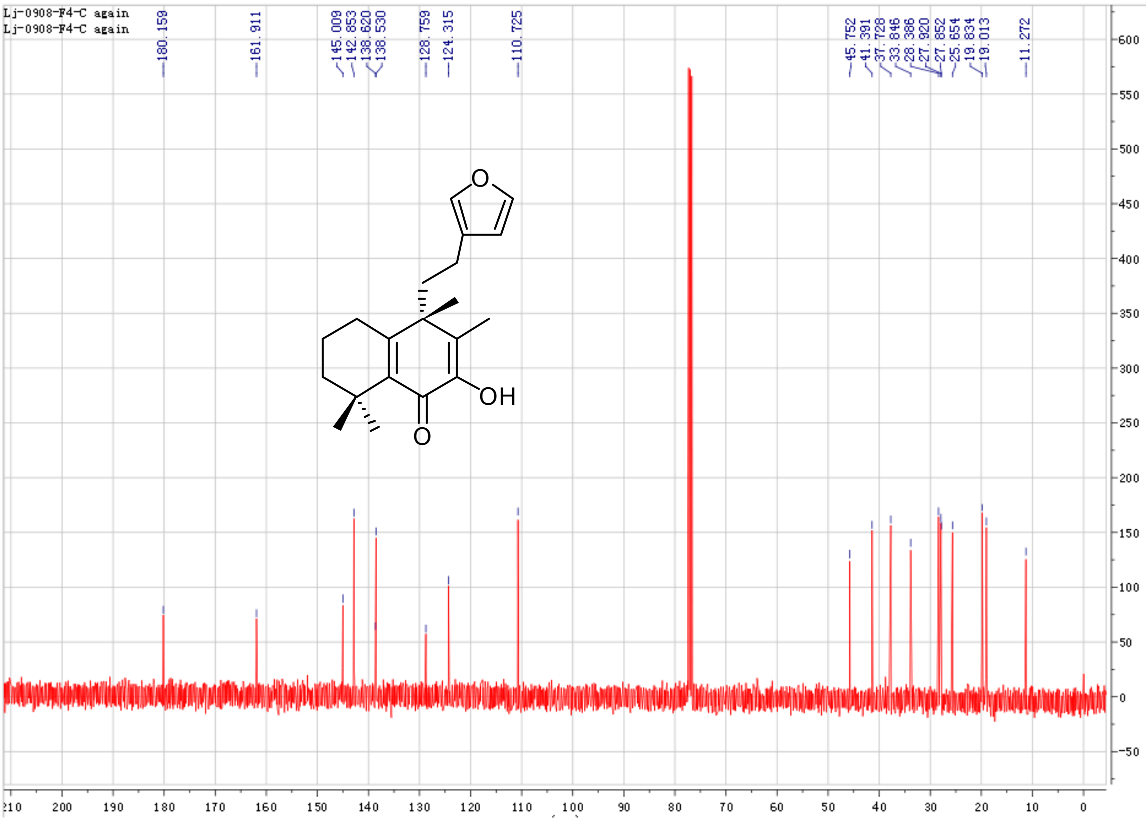

Figure S2. ^{13}C-NMR spectrum for isoleojaponin (**1**) in $CDCl_3$.

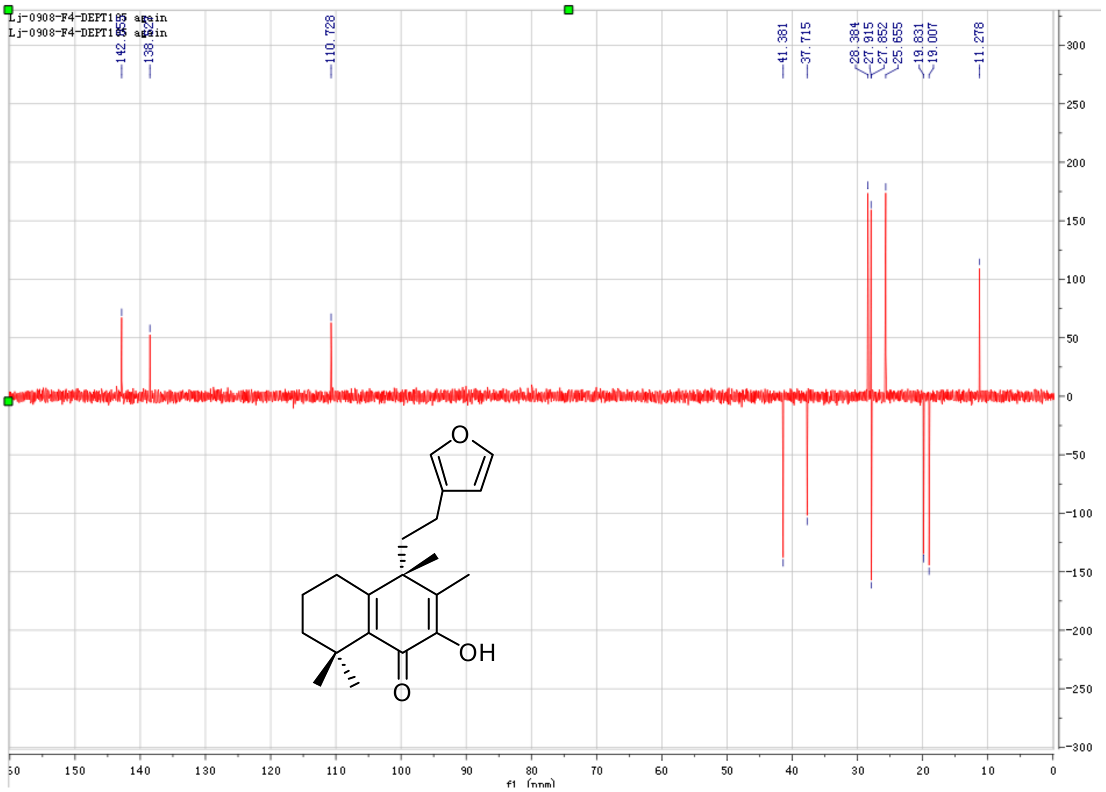

Figure S3. DEPT135 spectrum for isoleojaponin (**1**) in $CDCl_3$.

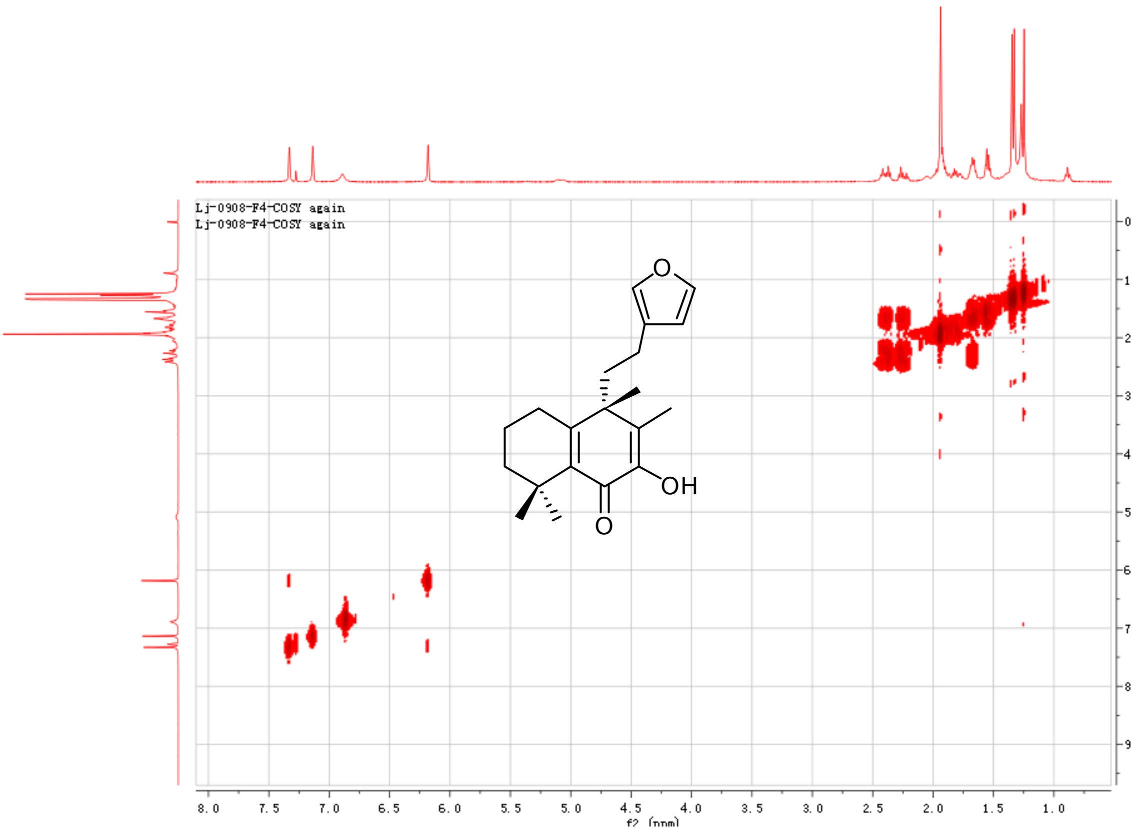

Figure S4. COSY spectrum for isoleojaponin (**1**) in $CDCl_3$.

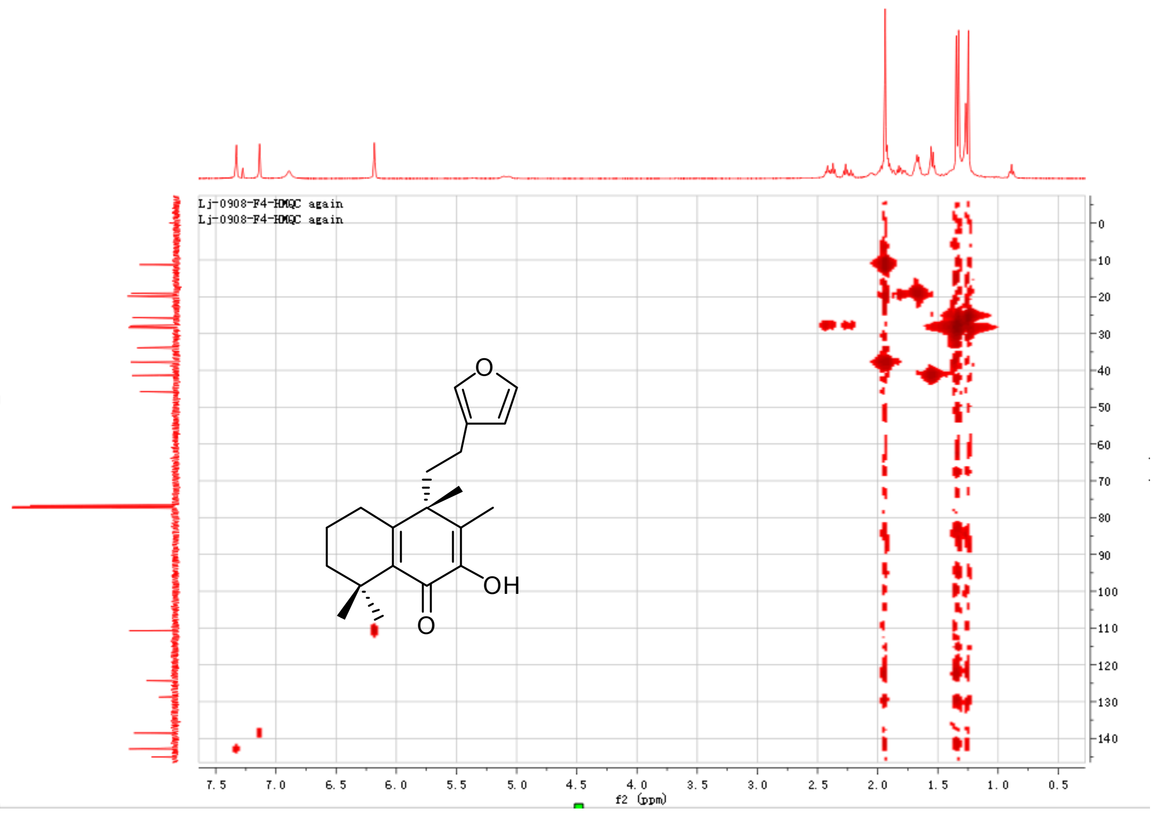

Figure S5. HMQC spectrum for isoleojaponin (**1**) in $CDCl_3$.

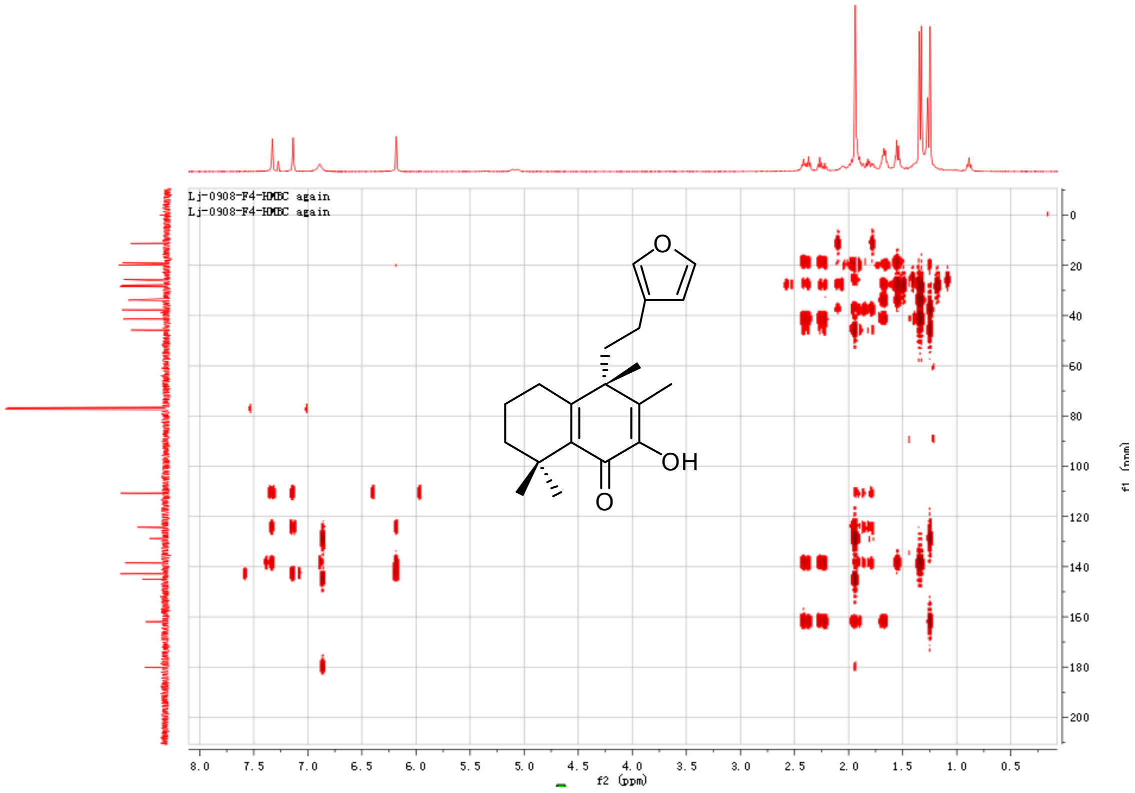

Figure S6. HMBC spectrum for isoleojaponin (**1**) in $CDCl_3$.

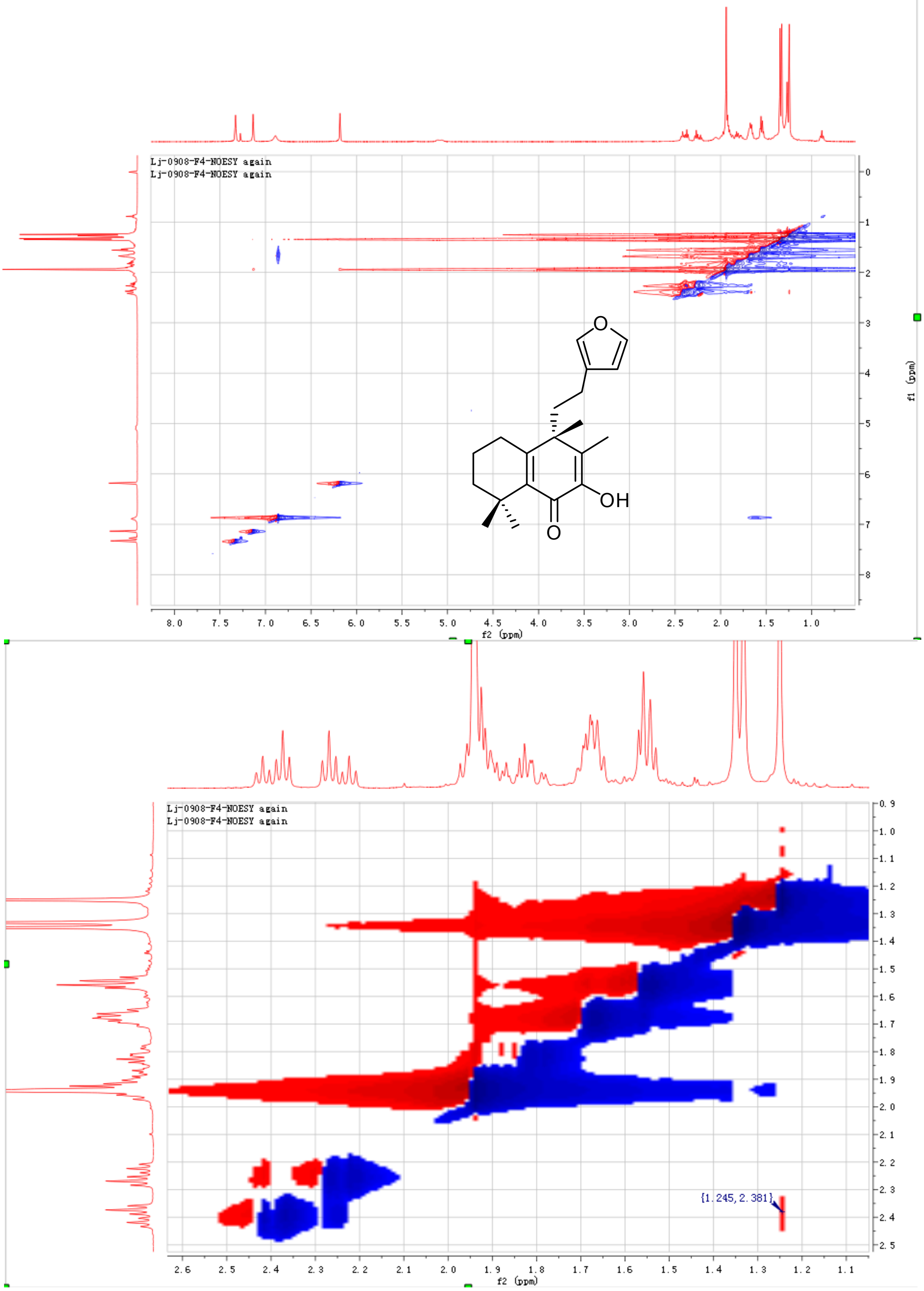

Figure S7. NOESY spectrum for isoleojaponin (**1**) in $CDCl_3$.

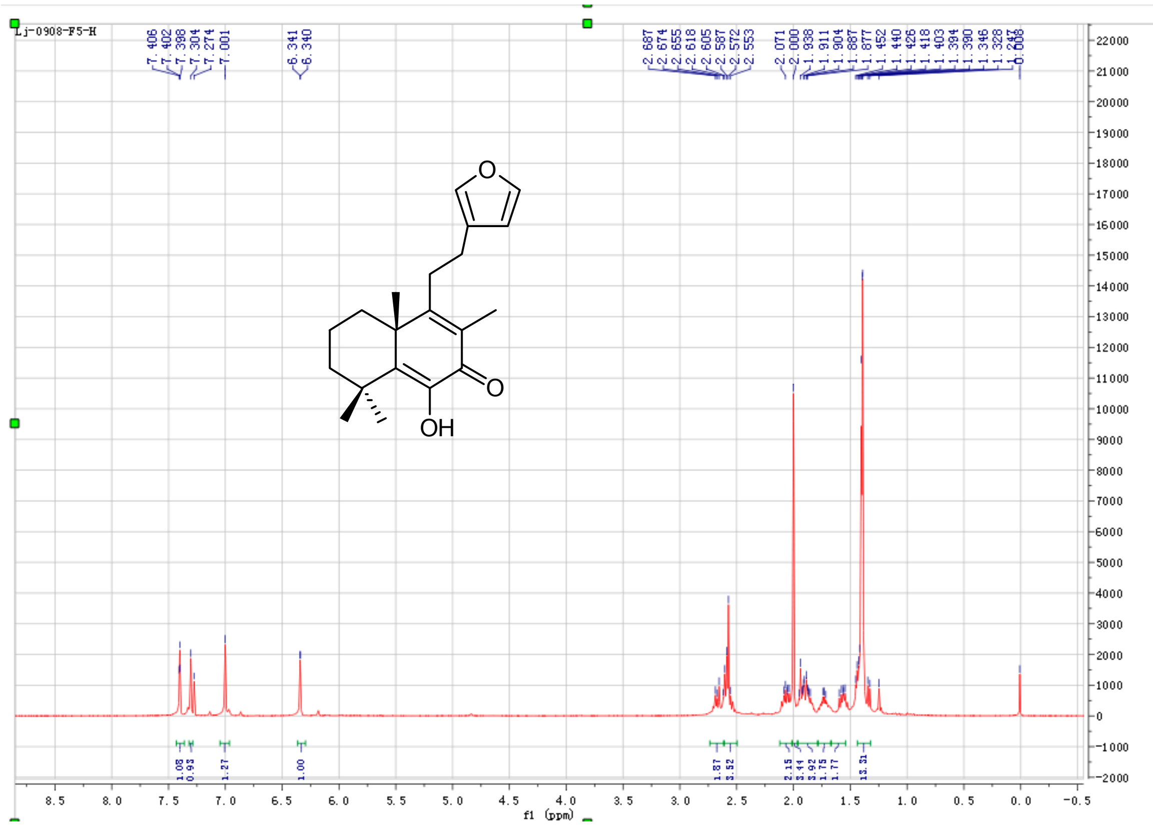

Figure S8. ^{1}H-NMR spectrum for leojaponin (**2**) in $CDCl_3$.

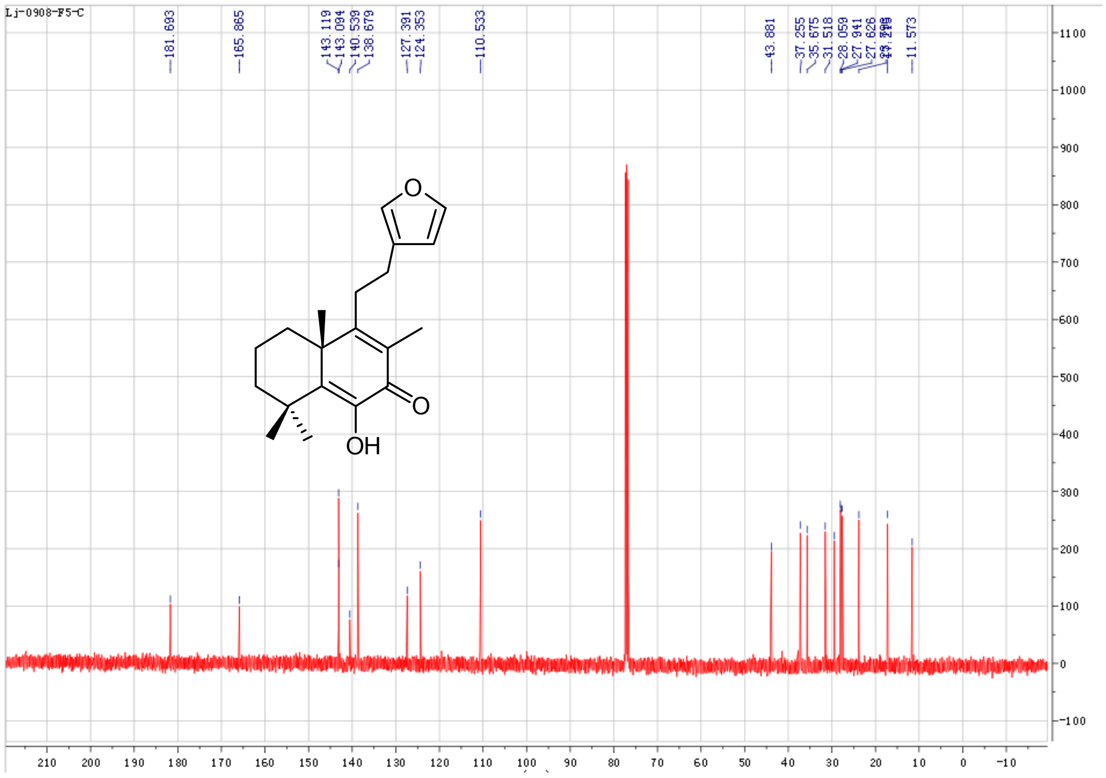

Figure S9. ^{13}C-NMR spectrum for leojaponin (**2**) in $CDCl_3$.

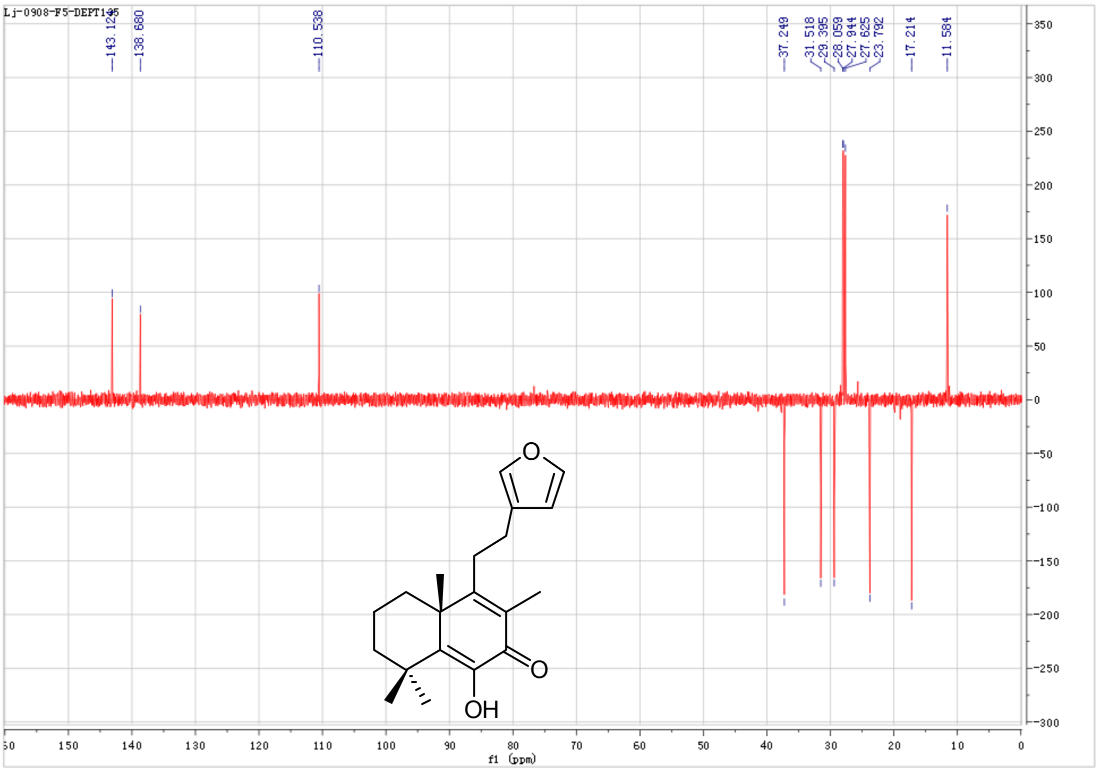

Figure S10. DEPT135 spectrum for leojaponin (**2**) in $CDCl_3$.
